# Treatment Monitoring of Carpal Tunnel Syndrome Using Shear Wave Elastography: A Pilot In Vivo Study

**DOI:** 10.7759/cureus.102587

**Published:** 2026-01-29

**Authors:** Annika N Hiredesai, Casey J Martinez, Nathaniel B Hinckley, Camryn S Payne, Nirvikar Dahiya, Azra Alizad, Kevin Renfree

**Affiliations:** 1 Department of Orthopedic Surgery, Mayo Clinic Alix School of Medicine, Phoenix, USA; 2 Department of Orthopedic Surgery, Mayo Clinic, Phoenix, USA; 3 Department of Radiology, Mayo Clinic, Phoenix, USA; 4 Department of Radiology, Mayo Clinic, Rochester, USA

**Keywords:** carpal tunnel syndrome, corticosteroid injection, median nerve, shear wave elastography, ultrasound

## Abstract

Purpose: Shear wave elastography (SWE) is a non-invasive technique for assessing median nerve (MN) stiffness in carpal tunnel syndrome (CTS). This study evaluated whether SWE measurements of the MN improve after corticosteroid injection (CSI) and correlate with symptom improvement in CTS.

Methods: Ten patients (18 wrists) with CTS underwent SWE, grip strength testing, and completed questionnaires before and six weeks after ultrasound-guided CSI. SWE measurements included pressure and velocity in longitudinal/transverse planes at two locations: proximal to and at the carpal tunnel. Progression to carpal tunnel release (CTR) was recorded. Multivariate analyses assessed associations between SWE and symptom scores, controlling for confounders.

Results: Functional Status Scale (FSS) and Symptom Severity Scale (SSS) scores improved post-injection (p=0.007 and p<0.001, respectively). Significant changes occurred in SWE carpal tunnel pressure and velocity (p<0.05). No significant association between SWE and CTR progression. Post-injection proximal MN pressure (p=0.005) and velocity (p=0.006) measured longitudinally were associated with FSS scores.

Conclusion: SWE measurements change with CSI but do not consistently predict symptom relief or surgery need. Questionnaires on daily function and symptoms may be more reliable for outcome tracking.

## Introduction

Carpal tunnel syndrome (CTS) is the most commonly diagnosed peripheral compression neuropathy, with a prevalence of 3.2% in the US population [1]. The mechanism of CTS is largely attributed to the compression of the median nerve (MN) as it passes from the forearm to the palm through a space-limited canal [2,3]. The true pathophysiology of this chronic compressive neuropathy is complex and incompletely understood, but likely involves elements of ischemia, fibrosis/scarring, intraneural edema, and shear stress [4,5]. Demyelination is thought to occur early, followed by axonal loss later in the disease process. An obvious inability to histologically examine chronically compressed human nerves limits our understanding of this condition [6]. Patients often present with a wide range of symptoms, including pain, numbness, tingling, weakness, and a loss of dexterity, and presentation can vary by patient age, symptom duration, and symptom severity [1]. The effect of symptom duration on structural nerve damage or treatment outcome is unknown, as is whether symptom severity and characterization are related to the structural and physiologic condition of the MN [1].

The diagnosis of CTS is typically based on clinical findings and electrodiagnostic testing (EDX), which can be painful for the patient [7,8]. Moreover, EDX results can be influenced by skin temperature, have a high false positive rate (43%), and lack a national reference standard [9]. Ultrasonography and, most recently, ultrasound (US) elastography techniques are noninvasive, widely available, and affordable imaging modalities used for diagnosing CTS. Strain elastography is a commonly used method, though it is qualitative and operator-dependent. Shear wave elastography (SWE) has also been used to assess the stiffness of MN and is being investigated as an alternative diagnostic tool [10-13]. Based on the propagation speed of waves induced by the acoustic radiation force, SWE utilizes a highly focused US beam aimed at the area of interest within tissue [14]. 

The treatment of CTS often involves injections or surgical decompression. Local corticosteroid injections (CSI) can provide short-term symptom relief and delay surgery for up to one year [15]. Response to CSI has high sensitivity and a positive predictive value for successful revision carpal tunnel release (CTR) [16]. The mechanism of action of CSI for CTS is unknown, as tenosynovial biopsies in these patients have failed to demonstrate inflammatory changes histologically [17]. Patients who have experienced severe, prolonged compression may develop intraneural fibrosis or axonal loss with a more limited response [18]. Patient age, symptom duration or severity, or metabolic comorbidities may negatively impact remyelination and improvement in symptoms [19, 20]. Symptom recovery can also be variable, with pain relief occurring quickly and predictably, but return of sensation occurring less predictably and often over a prolonged period [21].

To date, there have not been any well-designed studies attempting to correlate treatment outcomes for CSI with pretreatment diagnostic testing. Shear wave elastography is a non-invasive test that can objectively assess the physical properties of MN pre- and post-injection. There are a few studies demonstrating the use of SWE for CTS diagnosis, but other aspects of CTS, including the assessment of disease severity and treatment outcomes, have not been studied [13,22,23]. The aim of this pilot study was to assess whether SWE measurements of the MN (1) improve at six weeks following a therapeutic CSI in the carpal tunnel and (2) correlate directly with improvements in symptom severity and functional status outcomes after CSI.

## Materials and methods

Ethics statement

All data used in this study were de-identified and Health Insurance Portability and Accountability Act (HIPAA) compliant. Thus, ethical approval from our institutional review board was deemed exempt.

Study population and clinical assessment

This single-institution pilot study was conducted in an academic hand surgery practice and included 10 patients (18 wrists). Inclusion criteria consisted of a CTS-6 score >12 and a proximal median nerve (MN) cross-sectional area (CSA) >10 mm² measured by ultrasound.

Baseline clinical evaluation included grip strength testing using a second-level Jamar hydraulic hand dynamometer (Patterson Medical, Warrenville, IL) and lateral pinch strength using a Jamar hydraulic pinch gauge (Patterson Medical). Patients also completed the Symptom Severity Scale (SSS) and Functional Status Scale (FSS) components of the Carpal Tunnel Questionnaire (CTQ) [24].

Ultrasound and shear wave elastography acquisition

All ultrasound examinations were performed using GE LOGIQ™ E10 ultrasound systems (GE Healthcare, Wauwatosa, WI) equipped with shear wave elastography (SWE) capabilities, using an L8-18i linear array transducer (8-18 MHz). Initial grayscale B-mode imaging was performed to identify the median nerve anatomy and course. Longitudinal and transverse images of the median nerve were obtained at two standardized locations: proximal to the carpal tunnel and at the level of the carpal tunnel. Transverse images at the carpal tunnel were used to measure the median nerve CSA.

Following B-mode assessment, the system was switched to SWE mode. SWE acquisitions were obtained in both longitudinal and transverse planes at each of the two predefined locations (proximal to the carpal tunnel and at the carpal tunnel). To improve measurement reliability and minimize technical bias, the following standardized acquisition protocol was used. Copious ultrasound gel was applied, and the transducer was held as lightly as possible while maintaining adequate skin contact. Care was taken to avoid compression of the median nerve or surrounding tissues, as transducer pressure is known to artificially increase measured shear wave speed. In longitudinal acquisitions, the transducer was adjusted to ensure the median nerve appeared straight, flat, and parallel to the transducer face. In transverse acquisitions, care was taken to center the nerve within the field of view. Minor transducer tilting was performed to optimize nerve echogenicity on B-mode imaging, ensuring a bright and well-defined nerve appearance prior to SWE acquisition and to avoid anisotropy artifact. At least three SWE acquisitions were performed for each plane (longitudinal and transverse) at each anatomical location. Shear wave velocity and pressure measurements were recorded for each acquisition, and mean values were calculated for analysis. A real-time confidence map (color map) that dynamically updates was utilized on the equipment to ascertain that acquisition of data was taken from zones that produced high-quality, stable signals.

Post-injection follow-up

After completion of baseline imaging, patients underwent ultrasound-guided corticosteroid injection into the carpal tunnel consisting of 3 mL of 1% lidocaine and 1 mL (40 mg) of methylprednisolone acetate. At 6-week follow-up, patients underwent repeat ultrasound and SWE using the same acquisition protocol, along with repeat grip strength, pinch strength, and CTQ (FSS and SSS) assessments. Progression to CTR following injection was recorded and defined as treatment failure, which served as the primary study outcome. Changes in FSS and SSS scores were designated as secondary outcomes.

Statistical analysis

Continuous variables were described using mean and standard deviation (SD). Categorical variables were summarized using frequencies and proportions. Differences in pre- and post-injection measurements were summarized and assessed using the Wilcoxon signed-rank test. Multivariate logistic regressions were used to assess the association between post-injection SWE measurements, both baseline and differences from pre-injection, and treatment failure with covariates accounting for age, sex, BMI, diabetes mellitus, and thyroid disorders - all factors having previously been associated with CTS development and/or severity. Multivariate linear regressions were used to assess the association between post-injection SWE measurements, both baseline and differences from pre-injection, and post-injection differences in FSS and SSS scores, accounting for age, sex, BMI, diabetes mellitus, and thyroid disorders. A p-value less than 0.05 was considered statistically significant. All analyses were carried out using R software (R Foundation for Statistical Computing, Vienna, Austria).

## Results

A total of 18 wrists in 10 patients (8 female, 80%) were identified and enrolled in this study. The average patient age was 60.3 ± 13.0 years, and BMI was 33.2 ± 5.3. Patient comorbidities by number of wrists included diabetes mellitus (n=2, 11%), thyroid disorder (n=4, 22%), osteoarthritis of the hand or wrist (n=8, 44%), depression (n=17, 94%), and a systemic inflammation disorder (n=8, 44%). Bilateral CTS affected 80% of patients, and the average symptom chronicity prior to CSI was 5.8 ± 3.9 months. Six wrists (five patients) progressed to CTR after CSI (33%).

Table 1 illustrates average pre-injection and six weeks post-injection grip, pinch, FSS, and SSS. FSS and SSS scores were significantly lower post-injection (p=0.007 and p < 0.001, respectively). Table 2 illustrates the average pre-injection and six weeks post-injection MN proximal and carpal tunnel cross-sectional area (mm^2^), and MN anteroposterior (AP) diameter (mm). The reported difference value represents the mean of the differences calculated for each wrist.

**Table 1:** Means and differences in pre- and six-week post-injection grip strength, pinch strength, and Boston Carpal Tunnel Questionnaire subscores FSS, Functional Status Scale; SSS, Symptom Severity Scale

Parameter	Pre-injection	Post-injection	Difference	P-value
Grip Strength	21.5 ± 5.5	27.3 ± 10.8	4.5 ± 12.3	0.307
Pinch Strength	12.6 ± 3.6	15.6 ± 4.7	1.9 ± 5.9	0.503
FSS	18.8 ± 3.6	13.3 ± 6.7	5.5 ± 6.2	0.007
SSS	35.7 ± 7.4	20.6 ± 10.1	15.1 ± 1	<0.001

**Table 2:** Means and differences in pre- and six-week post-injection median nerve CSA and anteroposterior diameter measurements. CSA: cross-sectional area

Parameter	Location	Pre-injection	Post-injection	Difference	P-value
Median nerve cross-sectional area (mm^2^)	Proximal	8.0 ± 2.1	8.0 ± 1.5	-0.2 ± 1.6	0.830
	Carpal Tunnel	14.5 ± 4.5	15.1 ± 5.6	0.4 ± 2.4	0.792
Median nerve anteroposterior diameter (mm)	Proximal	2.5 ± 0.6	2.6 ± 0.5	0.1 ± 0.6	0.751
	Carpal Tunnel	2.4 ± 2.3	1.9 ± 0.4	0.1 ± 0.6	0.474

Average pre-injection proximal and carpal tunnel MN AP diameters were 2.5 ± 0.6 mm and 2.4 ± 2.3 mm, respectively. Average post-injection proximal and carpal tunnel MN AP diameters were 2.6 ± 0.5 mm and 1.9 ± 0.4 mm, respectively. When stratified by those who progressed to CTR and those who were successfully managed with CSI, average post-injection proximal (2.6 ± 0.5 mm vs. 2.4 ± 0.5 mm, p=0.621) and carpal tunnel (1.8 ± 0.3 mm vs. 2.1 ± 0.5 mm, p=0.190) MN AP diameters were not significantly different between groups. When comparing those who progressed to CTR and those who did not, average post-injection proximal (8.5 ± 1.7 mm^2^ vs. 7.3 ± 0.9 mm^2^, p=0.845) and carpal tunnel (17.1 ± 6.9 mm^2^ vs. 12.4 ± 1.8 mm^2^, p=0.407) MN CSAs were not significantly different between groups.

Table 3 illustrates median nerve pressure and velocity SWE measurements obtained pre- and 6 weeks post-injection. Average pre-injection MN proximal transverse and longitudinal pressures were 33.7 ± 18.8 kPa and 68.5 ± 40.5 kPa, respectively. Average pre-injection MN proximal transverse and longitudinal velocities were 3.2 ± 0.8 mm/s and 4.5 ± 1.5 mm/s, respectively. Average pre-injection MN carpal tunnel transverse and longitudinal pressures were 70.7 ± 54.2 kPa and 133.1 ± 84.2 kPa, respectively. Average pre-injection MN carpal tunnel transverse and longitudinal velocities were 4.5 ± 1.7 mm/s and 6.3 ± 2.2 mm/s, respectively. Average post-injection MN proximal transverse and longitudinal pressures were 32.0 ± 22.0 kPa and 58.8 ± 28.6 kPa, respectively. Average post-injection MN proximal transverse and longitudinal velocities were 3.2 ± 0.9 mm/s and 4.5 ± 1.1 mm/s, respectively. Average post-injection MN carpal tunnel transverse and longitudinal pressures were 94.2 ± 155.2 kPa and 114.0 ± 57.7 kPa, respectively. Average post-injection MN carpal tunnel transverse and longitudinal velocities were 4.2 ± 1.2 mm/s and 5.9 ± 1.5 mm/s, respectively. There were significant differences between pre- and post-injection SWE transverse and longitudinal measurements for carpal tunnel pressure and velocity. When stratified by those who progressed to CTR and those who responded favorably to CSI, average post-injection measurements were not significantly different between groups for all recorded SWE measurements. Similarly, there were no significant differences between differences in pre- and post-injection measurements when comparing those who achieved adequate symptom relief with CSI and those who progressed to CTR.

**Table 3:** Means and differences in pre- and six-week post-injection SWE measurements. SWE, shear wave elastography

Parameter	Location	Pre-injection	Post-injection	Difference	P-value
Median nerve pressure (kPa)	Proximal transverse	33.7 ± 18.8	32.0 ± 22.0	-1.7 ± 23.0	0.828
	Carpal tunnel transverse	70.7 ± 54.2	94.2 ± 155.2	43.4 ± 76.6	0.017
	Proximal longitudinal	68.5 ± 40.5	58.8 ± 28.6	-9.7 ± 57.1	0.433
	Carpal tunnel longitudinal	133.1 ± 84.2	114.0 ± 57.7	-101.1 ± 86.3	<0.001
Median nerve velocity (mm/s)	Proximal transverse	3.2 ± 0.8	3.2 ± 0.9	0.0 ± 1.0	0.948
	Carpal tunnel transverse	4.5 ± 1.7	4.2 ± 1.2	1.4 ± 2.4	0.029
	Proximal longitudinal	4.5 ± 1.5	4.5 ± 1.1	0.0 ± 1.9	0.813
	Carpal tunnel longitudinal	6.3 ± 2.2	5.9 ± 1.5	-3.1 ± 2.3	<0.001

Multivariate logistic regression controlling for age, sex, BMI, diabetes mellitus, and thyroid disorders status assessed whether any recorded post-injection SWE measurements or differences between pre- and post-injection SWE measurements were associated with CTR (Table 4). There were no significant associations between any recorded SWE measurements or differences in measurements before and after CSI and CTR. Multivariate linear regression controlling for the same covariates as the logistic regression assessed whether any post-injection SWE measurements or differences between pre- and post-injection SWE measurements were associated with post-treatment changes in FSS and SSS scores (Tables 5-6). There were no significant associations between any recorded SWE measurements or differences between pre- and post-injection and FSS or SSS scores, except for post-injection proximal MN pressure and velocity measured longitudinally. Proximal MN pressure (9.631, p=0.005) and velocity (0.800, p=0.006) were directly associated with FSS scores.

**Table 4:** Multivariate logistic regression results for SWE measurements and progression to CTR. SWE, shear wave elastography; CTR: carpal tunnel release; NULL, results not available due to regression convergence.

SWE Measurement	Location	Regression Estimate	P-value
Median nerve pressure (kPa)	Proximal transverse	0.049	0.242
	Carpal tunnel transverse	0.049	0.242
	Proximal longitudinal	0.008	0.742
	Carpal tunnel longitudinal	-0.002	0.900
Median nerve velocity (mm/s)	Proximal transverse	0.491	0.566
	Carpal tunnel transverse	0.491	0.566
	Proximal longitudinal	0.181	0.813
	Carpal tunnel longitudinal	-0.203	0.716
Difference in median nerve pressure (kPa)	Proximal transverse	-0.082	0.239
	Carpal tunnel transverse	NULL	NULL
	Proximal longitudinal	-0.007	0.580
	Carpal tunnel longitudinal	-0.015	0.484
Difference in median nerve velocity (mm/s)	Proximal transverse	-0.571	0.508
	Carpal tunnel transverse	NULL	NULL
	Proximal longitudinal	-0.923	0.279
	Carpal tunnel longitudinal	-0.525	0.447

**Table 5:** Multivariate linear regression results for SWE measurements and FSS scores SWE, shear wave elastography; FSS, Functional Status Scale.

SWE Measurement	Location	Regression Estimate	P-value
Median nerve pressure (kPa)	Proximal transverse	-0.060	0.305
	Carpal tunnel transverse	-0.060	0.305
	Proximal longitudinal	9.631	0.005
	Carpal tunnel longitudinal	0.008	0.727
Median nerve velocity (mm/s)	Proximal transverse	-0.847	0.524
	Carpal tunnel transverse	-0.847	0.524
	Proximal longitudinal	0.800	0.006
	Carpal tunnel longitudinal	0.462	0.586
Difference in median nerve pressure (kPa)	Proximal transverse	0.024	0.701
	Carpal tunnel transverse	0.030	0.057
	Proximal longitudinal	-0.005	0.821
	Carpal tunnel longitudinal	-0.005	0.760
Difference in median nerve velocity (mm/s)	Proximal transverse	0.392	0.755
	Carpal tunnel transverse	0.819	0.102
	Proximal longitudinal	-0.138	0.836
	Carpal tunnel longitudinal	-0.228	0.724

**Table 6:** Multivariate linear regression results for SWE measurements and SSS scores SWE, shear wave elastography; SSS, Symptom Severity Scale.

SWE Measurement	Location	Regression Estimate	P-value
Median nerve pressure (kPa)	Proximal transverse	-0.018	0.892
	Carpal tunnel transverse	-0.018	0.892
	Proximal longitudinal	0.116	0.157
	Carpal tunnel longitudinal	0.015	0.760
Median nerve velocity (mm/s)	Proximal transverse	1.355	0.644
	Carpal tunnel transverse	1.355	0.644
	Proximal longitudinal	3.395	0.152
	Carpal tunnel longitudinal	1.176	0.527
Difference in median nerve pressure (kPa)	Proximal transverse	-0.011	0.938
	Carpal tunnel transverse	0.037	0.317
	Proximal longitudinal	0.013	0.781
	Carpal tunnel longitudinal	-0.028	0.453
Difference in median nerve velocity (mm/s)	Proximal transverse	-1.160	0.673
	Carpal tunnel transverse	0.772	0.505
	Proximal longitudinal	0.309	0.832
	Carpal tunnel longitudinal	-0.854	0.544

## Discussion

The main finding of this study was that there were no significant detectable morphologic differences in the MN (SWE or US) in patients who underwent CSI and achieved adequate symptom relief compared to those who progressed to CTR. Secondarily, while there were direct associations between longitudinally-measured post-injection proximal MN pressure and velocity and FSS scores, no other post-injection MN measurements or differences in pre- and post-injection measurements were associated with treatment failure or FSS and SSS scores. Nevertheless, there was evidence that CSI did significantly impact SWE measurements, as there were significant differences in pre- and post-injection carpal tunnel median nerve elastography and velocity measurements when measured both transversely and longitudinally.

This study’s findings suggest that while CSI may result in some MN morphologic changes detectable by SWE, these changes are not associated with odds of treatment failure (CTR) and have very limited association with post-injection symptom severity. Currently, SWE is the only clinically available method capable of quantitatively assessing soft tissue stiffness. SWE has been used to non-invasively assess the stiffness of the MN and can be used for diagnosing CTS. We are unaware of other studies that attempt to correlate SWE pretreatment diagnostic results with treatment outcomes, nor the influence of carpal tunnel CSI on MN morphology and symptom improvement. Though the results of this study suggest that SWE may have limited utility in predicting symptom relief and treatment failure after CSI, the FSS and SSS scores were found to be significantly lower. There is limited prior evidence assessing the utility of patient-reported outcome measures (PROMs) to predict progression to CTR, with Chen et al. reporting that CTQ scores alone were insufficient to predict progression to surgical intervention in a retrospective cohort of 200 CTS patients [25]. PROMs such as the CTQ should continue to be assessed in a highly powered cohort for their value in predicting CTS patient treatment courses and potentially set patient expectations.

Given the nature of pilot studies, there are several limitations that may be addressed in future investigations, including the limited sample size of 10 patients at a single institution due to limited funding and time frame. Methodologically, SWE in a superficial structure was technically challenging, and measurements were taken while using copious amounts of gel to avoid inadvertent stress to the MN. However, this increased the distance between the region of interest and the transducer, and in some instances, such as larger wrists, the transducer was placed on the skin itself, albeit with minimal pressure. It was difficult to obtain a position with minimal inherent tension in the surrounding muscles. Thus, most patients were imaged in the supine position without a towel under the wrist to minimize muscle tension, though this may have led to diminished MN visualization. Moreover, this study’s analysis only addressed outcomes six weeks post-injection. The possibility of remodeling or elasticity changes of the MN that develop over a longer timespan should be considered in future studies. Moreover, recruiting a more diverse patient population by age, sex, and pre-existing comorbidities, while following up at multiple time points after injection, will provide more comprehensive, generalizable results.

## Conclusions

The findings of this pilot study suggest that SWE measurements do change with CSI treatment for CTS patients but do not necessarily predict symptomatic relief and progression to surgery. FSS and SSS scores may have potential value in predicting CSI treatment failure and merit further exploration in a larger study. Moreover, these results emphasize the need for elucidating the pathophysiology of CTS and the effects of idiopathic CTS on the elasticity and morphologic changes to the MN. Particularly, the exploration of the impact of CTR on post-operative elasticity of the MN over time may be informative. The mechanisms by which CSI may provide relief in some patients, but not all, remain unclear and should also be considered in future investigations with longer-term follow-up. Overall, the results of this study may drive clinicians to explore alternative diagnostic tools to improve our understanding of prognostic indicators and, therefore, patient care outcomes in the setting of CTS.
